# Head-to-head comparison of myocardial perfusion SPECT and CMR for assessment of myocardial ischemia

**DOI:** 10.1186/1532-429X-17-S1-P119

**Published:** 2015-02-03

**Authors:** Fredrik Hedeer, Marcus Carlsson, Håkan Arheden, Henrik Engblom

**Affiliations:** Cardiac MR group Lund, Dept. of Clinical Physiology, Lund University, Lund, Sweden

## Background

Myocardial perfusion single photon emission computed tomography (MPS) and cardiac magnetic resonance (CMR) can be used for assessment of myocardial ischemia. The methods reveal different aspects of the pathophysiology associated with myocardial ischemia where MPS is based on uptake of a perfusion tracer in viable mitochondria whereas CMR is based on first-pass perfusion reflecting coronary in-flow kinetics. The aim of this study was to perform a head-to-head comparison between MPS and CMR for assessment of myocardial ischemia under identical perfusion conditions at stress.

## Methods

Ninety-six patients (34 females) were included in the study. All patients completed both gated MPS and CMR at rest and during adenosine stress. The MPS and CMR perfusion tracers were injected in the MR scanner during the same adenosine stress session. MPS images were acquired approximately one hour after the first-pass perfusion images. Qualitative image analysis for stress-induced ischemia was performed by two expert readers blinded to all clinical data.

## Results

**Figure 1:**
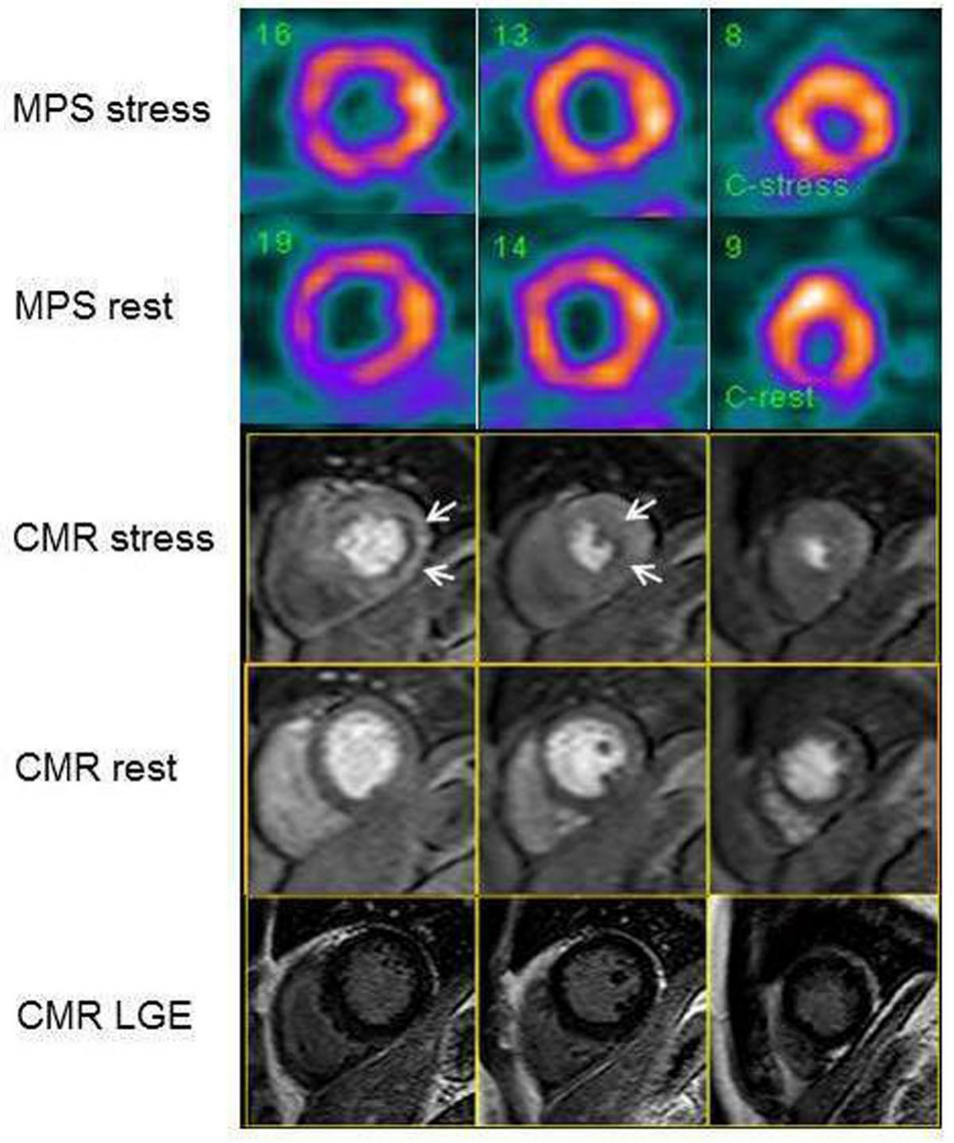
**The figure shows short-axis slices of myocardial perfusion at rest and stress with myocardial perfusion SPECT (MPS) and first-pass perfusion at rest and stress with cardiac magnetic resonance (CMR).** The MPS and CMR perfusion tracers were injected in the MR scanner during the same adenosine stress session. This is an example of a patient with no stress induced ischemia with MPS. CMR, however, shows stress induced ischemia in the subendocardial portion of the lateral wall (white arrows). CMR late gadolinium enhancement (LGE) images show no signs or myocardial infarction.

There were disagreements between MPS and CMR in 29% (28/96) of the patients. In 17 patients CMR showed signs of stress-induced ischemia with a normal MPS and in 11 patients the MPS was positive with no signs of stress-induced ischemia with CMR (Table 1). Figure 1 shows an example of a patient where CMR shows stress-induced ischemia but no ischemia is seen with MPS. The Cohen's kappa value for the agreement between MPS and CMR regarding stress-induced ischemia was 0.15.

**Table 1:** Number of patients with myocardial ischemia for MPS and CMR respectively.

	MPS +	MPS -
**CMR +**	**7 (7%)**	**17 (18%)**
**CMR -**	**11 (11%)**	**61 (64%)**

*CMR*, cardiac magnetic resonance; *MPS*, myocardial perfusion SPECT; + = ischemia; - = no ischemia.

## Conclusions

When assessing myocardial ischemia in patients under identical myocardial perfusion conditions at stress there are disagreements between MPS and CMR in a significant number of cases. The prognostic significance of these findings remains to be determined.

## Funding

N/A.

